# Enzymatic Synthesis of Isotopically Labeled Hydrogen Peroxide for Mass Spectrometry-Based Applications

**DOI:** 10.1021/jasms.4c00326

**Published:** 2024-10-04

**Authors:** Margaret Hoare, Ruiyue Tan, Isabella Militi, Kevin A. Welle, Kyle Swovick, Jennifer R. Hryhorenko, Sina Ghaemmaghami

**Affiliations:** † Department of Biology, University of Rochester, Rochester, New York 14627, United States; ‡ University of Rochester Mass Spectrometry Resource Laboratory, University of Rochester Medical Center, Rochester, New York 14627, United States

**Keywords:** Mass Spectrometry (MS), Glucose Oxidase (GOx), Methionine Oxidation

## Abstract

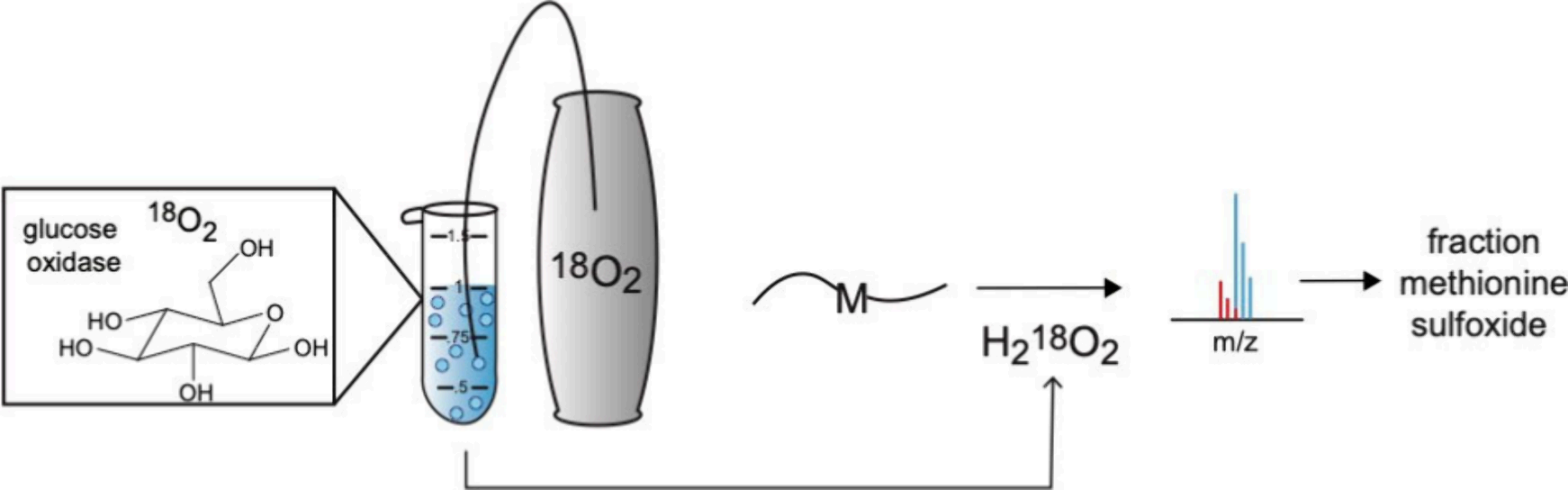

Methionine oxidation is involved in multiple biological processes including protein misfolding and enzyme regulation. However, it is often challenging to measure levels of methionine oxidation by mass spectrometry, in part due to the prevalence of artifactual oxidation that occurs during the sample preparation and ionization steps of typical proteomic workflows. Isotopically labeled hydrogen peroxide (H_2_^18^O_2_) can be used to block unoxidized methionines and enables accurate measurement of *in vivo* levels of methionine oxidation. However, H_2_^18^O_2_ is an expensive reagent that can be difficult to obtain from commercial sources. Here, we report a method for synthesizing H_2_^18^O_2_ in-house. Glucose oxidase catalyzes the oxidation of β-d-glucose and produces hydrogen peroxide in the process. We took advantage of this reaction to enzymatically synthesize H_2_^18^O_2_ from ^18^O_2_ and assessed its concentration, purity, and utility in measuring methionine oxidation levels by mass spectrometry.

## Introduction

Side chains of methionines are susceptible to oxidation by reactive oxygen species (ROS) or monooxygenases.^1−6^ This post-translational modification converts the nonpolar methionine residues (Met) to polar methionine sulfoxide residues (MetO).^1−4^ MetO formation can induce protein misfolding and has been linked to a number of neurodegenerative disorders and pathological aging.^1−4,6^ Additionally, regulated methionine oxidation can modulate diverse cellular processes and signaling pathways.^6^ Due to its involvement in protein damage and functional regulation, global quantification of methionine oxidation can provide important insights into a number of diverse biological processes.

It is challenging to measure levels of *in vivo* methionine oxidation by mass spectrometry in part because unoxidized methionines can become spontaneously oxidized during typical proteomic workflows.^1−4^ Selective blocking of unoxidized methionines can prevent this artifactual oxidation and allow for more accurate quantitation of methionine oxidation. An example of such an approach is methionine oxidation by blocking (MObB).^1−4^ In MObB, unoxidized methionines within denatured proteins are fully oxidized with isotopically labeled hydrogen peroxide (H_2_^18^O_2_) and blocked from spontaneous oxidation in subsequent steps of bottom-up proteomic workflows.^1,2^ Relative levels of ^16^O- and ^18^O-modified peptides can then be measured and used to determine levels of endogenously oxidized methionines.^1,2^ Furthermore, isotopically labeled hydrogen peroxide can be utilized to assess protein stability and protein–ligand binding using approaches such as stability of proteins from rates of oxidation (SPROX).^7^ In addition to its utility in quantifying methionine oxidation, H_2_^18^O_2_ has been employed in other mass spectrometric applications including quantitation of H_2_O_2_-producing reactions, analysis of the effects of H_2_O_2_ on metabolite synthesis, and quantitation of H_2_O_2-_induced oxidation of macromolecules.^8−10^

Despite its usefulness in diverse mass spectrometric applications, H_2_^18^O_2_ is expensive and can be difficult to obtain from commercial sources. For example, the sale of H_2_^18^O_2_ was entirely discontinued between 2021 and 2024, and currently there is only a single supplier for this reagent (Sigma). Traditionally, hydrogen peroxide is synthesized through the hydrogenation and subsequent oxidation of anthraquinone, an aromatic organic compound that acts as a catalyst in this reaction.^11,12^ In the most popular synthetic methods, anthraquinone is hydrogenated by a trickle bed with a palladium catalyst. The hydrogenated anthraquinone is then oxidized by O_2_ through a bubble column, restoring anthraquinone and producing hydrogen peroxide in the process.^11^ The synthesized hydrogen peroxide is extracted using a sieve-plate extraction tower before it is purified via distillation. Alternative synthesis methods involving electrodes, biochemical approaches, or electrosynthesis reactions with different catalysts have also been reported.^12^

Enzymatic synthesis provides a more practical approach for in-house generation of H_2_^18^O_2_ in typical biochemical laboratories. Glucose oxidase (GOx), an oxidoreductase that originates from insects and fungi, has been used in multiple industries including pharmaceuticals, food, textiles, and biofuels.^13−17^ GOx catalyzes the oxidation of β-d-glucose to d-glucono-δ-lactone using a FAD cofactor and generates H_2_O_2_ by reduction of molecular oxygen (O_2_).^5,13−15,17−19^ Previously, GOx from *Aspergillus niger* has been used to generate ∼11 mM H_2_O_2_ for use in textile bleaching studies.^16^ In this study, we have optimized this enzymatic reaction and used ^18^O_2_ as the substrate to generate ∼200 mM H_2_^18^O_2_ with high isotopic purity. We further demonstrated the efficacy of in-house generated H_2_^18^O_2_ in conducting quantitative mass spectrometric analyses of methionine oxidation levels.

## Results and Discussion

### Formation and Characterization of H_2_^18^O_2_ Generated by GOx

**Figure 1:**
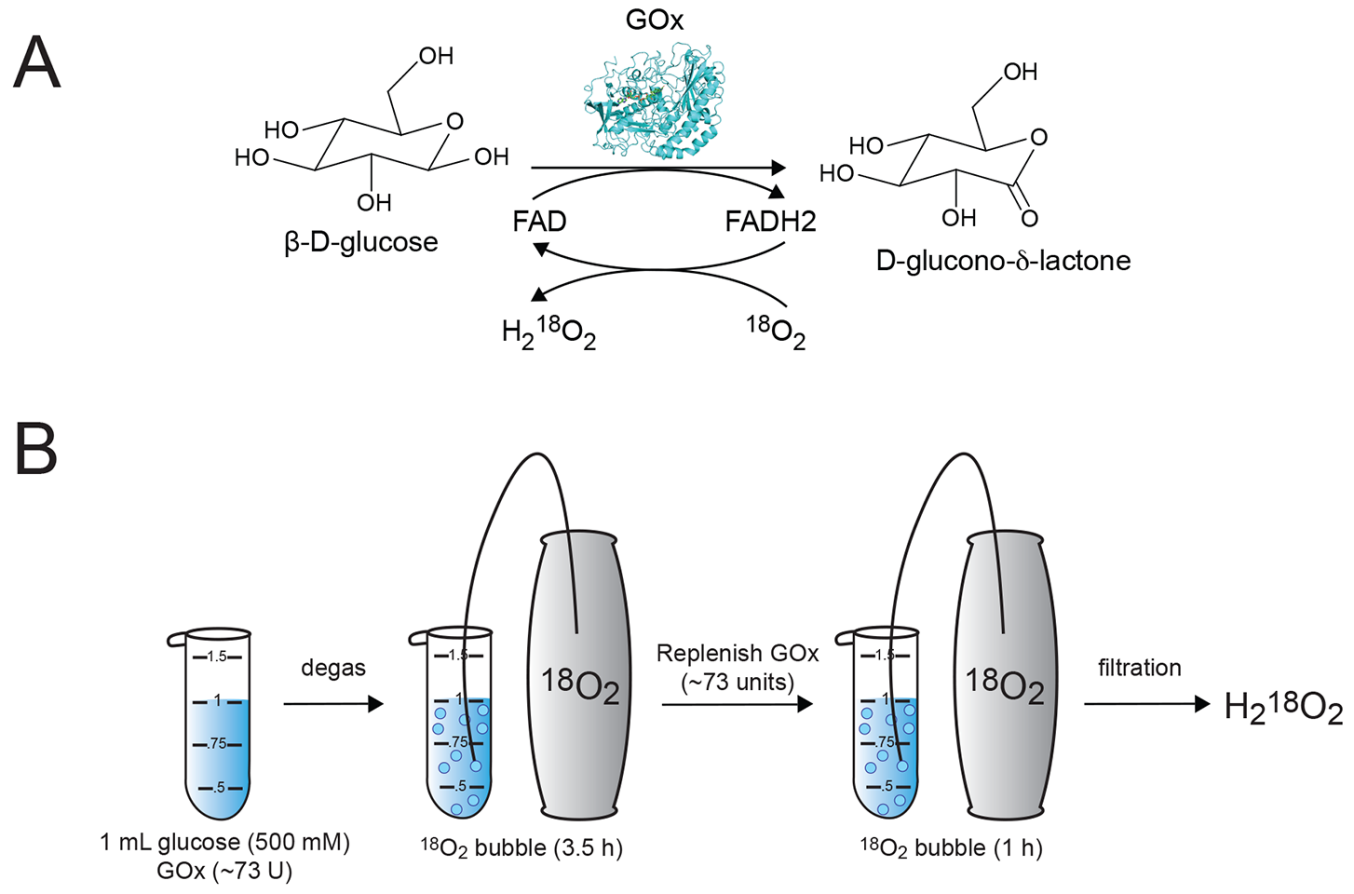
Reaction of glucose oxidase (PDB: 1GAL)^19^ with β-d-glucose and ^18^O_2_ generates H_2_^18^O_2_. (A) Reaction mechanism of glucose oxidase. (B) Experimental protocol used for generation of H_2_^18^O_2_. Details of the protocol are described in the Experimental Section.

The mechanism for production of hydrogen peroxide by glucose oxidase is illustrated in Figure 1A. We utilized this enzymatic reaction to synthesize H_2_^18^O_2_ from ^18^O_2_ as described in detail in the Experimental Section (Figure 1B). In brief, the enzymatic conversion of β-d-glucose to d-glucono-δ-lactone was carried out in the presence of a slow flow of ^18^O_2_ into an initially degassed solution containing GOx. In initial experiments we observed that the evolution of H_2_O_2_ deactivates GOx over time. Thus, to maximize the yield of H_2_O_2_, the reaction was supplemented with additional GOx after an initial generation period of 3.5 h, allowing further generation of H_2_O_2_ for an additional hour. Following the reaction, GOx was removed from the mixture by filtration.

**Figure 2:**
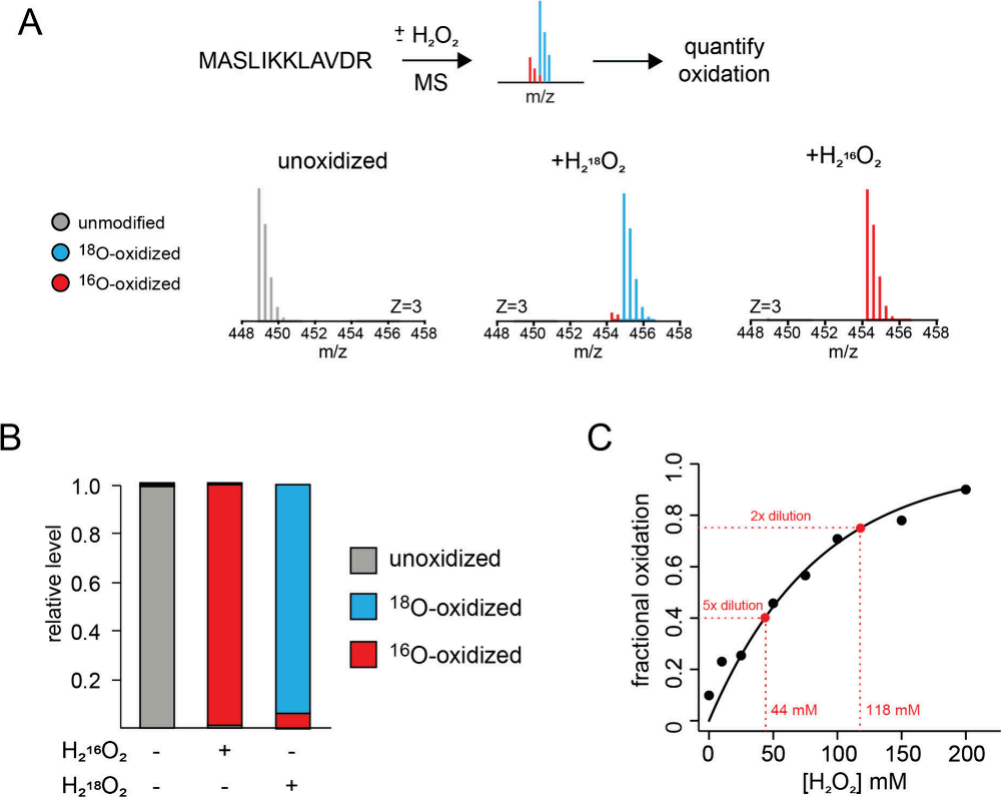
Synthesized H_2_^18^O_2_ has high isotopic purity and concentration. (A, B) Synthetic peptide was oxidized with the in-house generated H_2_^18^O_2_ (A) and shown to be 94% ^18^O-labeled (B). (C) Concentration of in-house generated H_2_^18^O_2_ was determined to be ∼230 mM by comparing its efficiency in oxidizing a model peptide with known concentrations of the H_2_^16^O_2_ ladder.

The purity and concentration of generated H_2_O_2_ was measured by mass spectrometry. An unoxidized synthetic peptide was fully oxidized with the generated H_2_^18^O_2_ or commercially obtained H_2_^16^O_2_ (Figure 2A). Relative levels of unmodified, ^16^O-modified and ^18^O-modified peptides were measured by mass spectrometry (Figure 2B). The peptide oxidized with in-house generated H_2_^18^O_2_ contained ∼94% ^18^O-labeled methionines, indicative of the isotopic purity of the oxidant. In a second experiment, the peptide was partially oxidized with known concentrations of H_2_^16^O_2_, and the resulting oxidation levels, as measured by mass spectrometry, were compared to peptides oxidized with various dilutions of in-house generated H_2_^18^O_2_. This comparison indicated that the in-house generated H_2_^18^O_2_ had a concentration of ∼230 mM (Figure 2C).

### Generated H_2_^18^O_2_ Can Be Used to Accurately Measure Methionine Oxidation Levels

**Figure 3:**
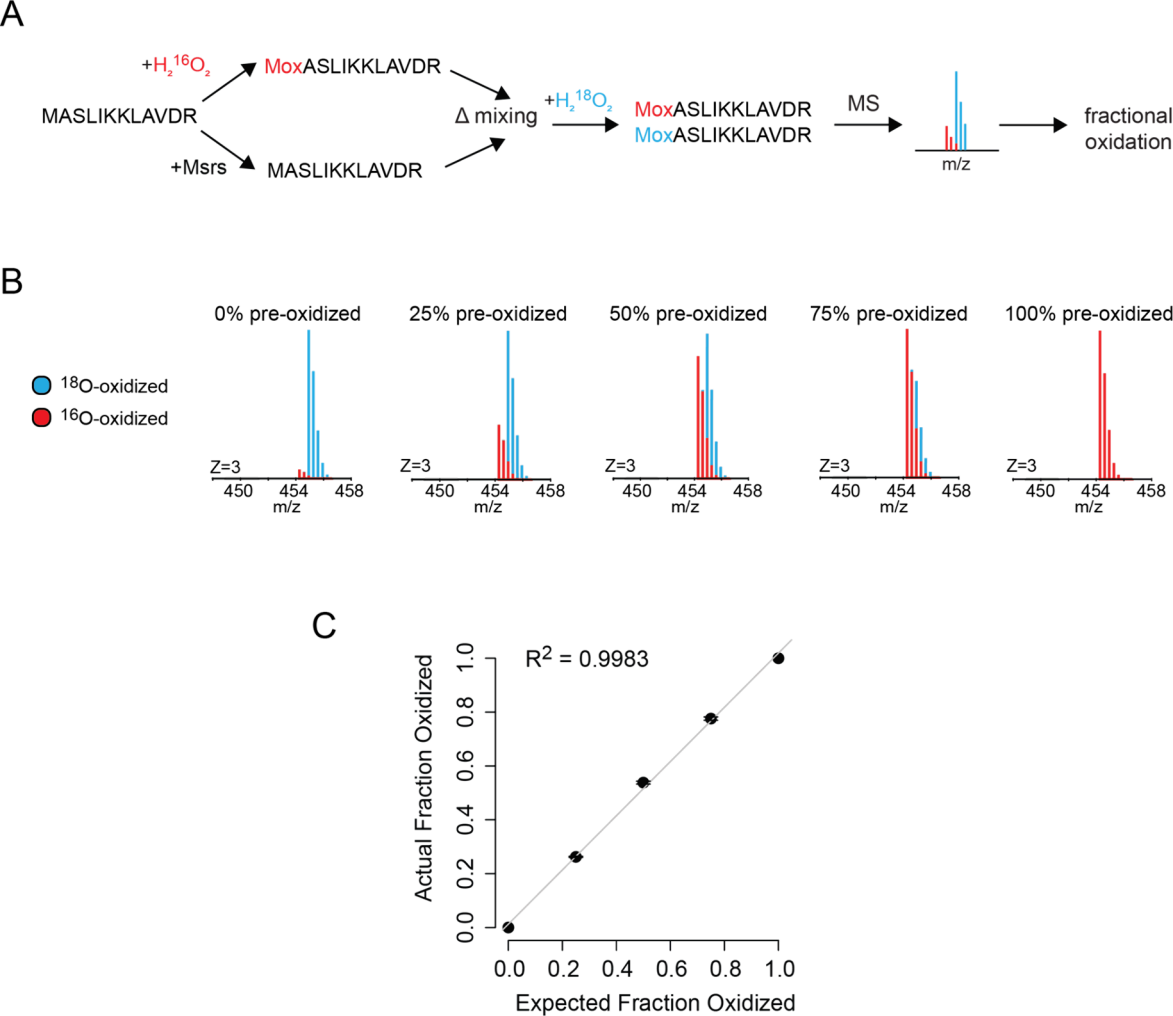
Synthesized H_2_^18^O_2_ can be used to accurately measure levels of methionine oxidation by mass spectrometry. (A, B) Preoxidized peptide mixtures containing different levels of ^16^O-methionines (0%, 25%, 50%, 75%, 100%) were fully oxidized with H_2_^18^O_2_ (A) and analyzed by mass spectrometry (B). (C) Fractional oxidation with ^16^O was measured and normalized to unoxidized and oxidized controls. The pairwise plot shows the correlation between measured and expected ^16^O-oxidation levels for each mixture. The error bars indicate standard deviations of two replicate experiments.

Next, we demonstrated that the generated H_2_^18^O_2_ can be used as an effective blocking agent, enabling the accurate measurement of methionine oxidation levels. A synthetic peptide was fully reduced by methionine sulfoxide reductase A and methionine sulfoxide reductase B (Msrs) or fully oxidized with H_2_^16^O_2_. Oxidized peptide was mixed with Msr-treated unoxidized peptide at variable ratios to generate mixtures with known predetermined methionine oxidation levels. These mixtures were then oxidized with diluted H_2_^18^O_2_, resulting in ^18^O-oxidation of the previously unoxidized methionines. Relative ^16^O-oxidation levels of each peptide mixture were determined by measuring the fractional populations of ^16^O- and ^18^O-oxidized peptides. The pairwise comparison of expected versus measured ^16^O-oxidation levels of peptide mixtures is shown in Figure 3. This analysis demonstrates that in-house generated H_2_^18^O_2_ can be effectively used as an isotopically labeled blocking reagent for accurate measurement of methionine oxidation levels.

In-house synthesis also provides a more cost-effective approach for obtaining ^18^O-labeled hydrogen peroxide (at the time of writing this manuscript, it reduced costs by approximately 50% in comparison to commercial sources). However, although the described method for in-house synthesis of hydrogen peroxide is straightforward and accessible, there are two important caveats that require special consideration. First, the isotopic purity of the H_2_^18^O_2_ produced is dependent on the purity of the dissolved ^18^O_2_ in the reaction buffer. Thus, to obtain isotopically pure H_2_^18^O_2_, removal of ^16^O_2_ by careful initial degassing, and subsequent use of highly pure ^18^O_2_ as a substrate is required. Second, the H_2_^18^O_2_ generated using the described protocol will also contain buffer components (in this case, sodium acetate) and glucose in oxidized and unoxidized forms. These impurities were inconsequential to the methionine blocking applications investigated in this study. However, if downstream applications require chemically pure H_2_^18^O_2_, further purification of the generated product may be required.

## Conclusions

This study describes a protocol that employs a widely available enzyme, glucose oxidase, for generation of concentrated and isotopically enriched ^18^O-labeled hydrogen peroxide. The synthesized H_2_^18^O_2_ can be used to block unoxidized methionines and facilitate the measurement of methionine oxidation levels in mass spectrometric workflows.

## Experimental Section

To generate H_2_^18^O_2_, 1.5 mL of H_2_^18^O (Cambridge Isotope Laboratories, OLM-240-10G) was degassed in a 2 mL Eppendorf tube inside of a sealed vacuum flask connected to a vacuum. Glucose and sodium acetate were added to 1 mL of H_2_^18^O to attain final concentrations of 500 mM and 50 mM, respectively. Activity units (72.8) of glucose oxidase (Sigma, G2133-10KU) was added to the solution, then ^18^O_2_ (Sigma, 602892-1L) was slowly bubbled from a pipet tip attached to tubing connected to a 1 L gas tank for 4.5 h at 35 °C. Note that one activity unit is defined as 1.0 μmole of hydrogen peroxide per minute at 35 °C and pH 5.1. After 3.5 h, another 72.8 units of glucose oxidase was added to the tube and the slow bubble of ^18^O_2_ continued for another hour. After incubation, the hydrogen peroxide was purified through centrifugation at 14,000*g* for 20 min at 4 °C in a 0.5 mL Amicon filter (3 kDa MWCO) to remove the glucose oxidase. The synthesized H_2_^18^O_2_ was aliquoted and stored at −20 °C until use. Additional experimental information related to the determination of the purity and concentration of H_2_^18^O_2_ and mass spectrometric analyses are provided in the Supporting Information.

## Supporting Information Available

Experimental details, including determination of the purity and concentration of H_2_^18^O_2_, generation of peptide mixtures with different oxidation levels, and mass spectrometry workflow (PDF)

## Abbreviations

ROS: reactive oxygen species
Msrs: methionine sulfoxide reductases
MObB: methionine oxidation by blocking
GOx: glucose oxidase
FAD: flavin adenine dinucleotide
H_2_O_2_: hydrogen peroxide
Met: methionine
MetO: methionine sulfoxide
