# Supplementary material for: Enzymatic Synthesis of Isotopically Labeled Hydrogen Peroxide for Mass Spectrometry-Based Applications
Source: J Am Soc Mass Spectrom. 2024 Oct 4;35(12):3308–12. doi: 10.1021/jasms.4c00326 (PMC11622373; doi:10.1021/jasms.4c00326)
Supplement: Supplementary file 1 — js4c00326_si_001.pdf [file js4c00326_si_001.pdf]

Supporting Information for:

Enzymatic synthesis of isotopically labeled hydrogen peroxide for mass spectrometry-based applications

Margaret Hoare[1], Ruiyue Tan[1], Isabella Militi[1], Kevin A. Welle[2], Kyle Swovick[2], Jennifer R. Hryhorenko[2], Sina Ghaemmaghami[1,2*]

[1]Department of Biology, University of Rochester, Rochester, NY, 14627, USA

[2]University of Rochester Mass Spectrometry Resource Laboratory, University of Rochester Medical Center, Rochester, NY, 14627, USA

[*]Correspondence:

Sina Ghaemmaghami
sina.ghaemmaghami@rochester.edu
585-275-4829,
326 Hutchison Hall
University of Rochester
Rochester NY 14627

Materials and methods

Determination of the purity and concentration of $H_2{}^{18}O_2$

To determine the purity of $H_2{}^{18}O_2$, 10 µg of a synthetic peptide (MASLIKKLAVDR) was incubated with diluted $H_2{}^{18}O_2$ at 37 °C for 30 minutes and compared to an unoxidized control. Samples were frozen and lyophilized then desalted through homemade C18 columns and eluted in 50/50% acetonitrile (ACN)/H_2O in 0.1% formic acid (FA). Samples were analyzed by direct injection mass spectrometry as described below. Raw MS data was analyzed using XCailbur (Thermo). Relative levels of unmodified, ^{16}O- and ^{18}O-oxidized peptides were determined by analyzing the intact mass spectra as described previously.[1]

To determine the concentration of $H_2{}^{18}O_2$, 10 µg of a synthetic peptide (MASLIKKLAVDR) was incubated with differing concentrations of $H_2{}^{16}O_2$ (Fisher Bioreagents) or a 2x or 5x dilution of the generated $H_2{}^{18}O_2$ solution for 10 minutes at 37 °C. Oxidation was quenched with 400 mM sodium sulfite and samples were frozen and lyophilized. Samples were desalted through homemade C18 columns and eluted in 50/50% ACN/H_2O in 0.1% FA and analyzed by direct injection mass spectrometry as described below. Fractional oxidation was determined by least squares fitting the ^{16}O-oxidation standard curve to a single exponential equation with the software KaleidaGraph and using the resulting fitted equation to determine the concentrations of the ^{18}O-oxidized peptides.

Generation of peptide mixtures with different oxidation levels

To generate fully oxidized peptides, 25 μg (372 μM) of MASLIKKLAVDR at a concentration of 0.5 mg/ml was oxidized with 160 mM H_2O_2 for 30 minutes at 37 °C. The sample was frozen and lyophilized to remove the hydrogen peroxide, then desalted in a homemade C18 column and eluted in 50% ACN/H_2O in 0.1% trifluoroacetic acid (TFA). To generate fully unoxidized peptides, 25 μg of the peptide was incubated with 50 mM dithiothreitol (Sigma), 1.25 μM of Methionine Sulfoxide Reductase A and 12.3 μM of Methionine Sulfoxide Reductase B that were recombinantly expressed and purified from *E.coli* [2,3] in 50 mM Tris buffer for 45 minutes at 37 °C. Samples were lyophilized then desalted to remove enzymes and salts. After desalting, fully oxidized and fully reduced peptides were mixed to generate specific fractional oxidations. These pre-oxidized mixtures were then frozen and lyophilized before reconstitution in 1.25x diluted $H_2{}^{18}O_2$. The peptides were oxidized for 30 minutes at 37 °C, then frozen and lyophilized before desalting with homemade C18 columns into 50/50% ACN/H_2O in 0.1% FA. The sample was diluted to 15 μg/mL before analysis by mass spectrometry.

Mass Spectrometry

Peptides were diluted in 50/50% ACN/H_2O in 0.1% FA to 15μg/mL. 40ul of this peptide was directly injected into a Q Exactive Plus Mass Spectrometer (Thermo Fisher) using a Dionex Ultimate 3000 HPLC (Thermo Fisher) with a flow rate of 100μL/min. The solvent was a 50/50% mixture of 0.1% FA in H_2O and 0.1% FA in ACN that was injected for 3 minutes. A HESI source set in positive mode ionized the peptides at resolution of 70,000

at m/z 200 with a 240 ms maximum injection time, AGC target of 1e6, and an overall range of 300-2000 m/z. MS1 files were generated from raw files using MSConvert[4] and fractional oxidations were measured as previously described.[1] Under some conditions (e.g. oxidation ladder experiments shown in figure 3 where fractional ^{16}O oxidation was high prior to blocking with ^{18}O), a population of the peptide (up to 33% in the fully oxidized sample) was oxidized to form methionine sulfones in addition to methionine sulfoxides. In these cases, it was noted that ratios of $^{16}O/^{18}O$-oxidized relative to $^{18}O/^{18}O$-oxidized sulfone-containing peptides and ratios of ^{16}O-oxidized relative to ^{18}O-oxidized sulfoxide-containing peptides were similar in magnitude and reflected the expected initial ^{16}O oxidation levels. In such cases, fractional oxidation levels were determined by measuring the average of the two ratios normalized by their relative intensities.

Abbreviations

ACN- acetonitrile

FA- formic acid

TFA- trifluoroacetic acid

HESI – heated electrospray ionization

AGC – automatic gain control

References

(1) Tan, R.; Hoare, M.; Welle, K. A.; Swovick, K.; Hryhorenko, J. R.; Ghaemmaghami, S. Folding Stabilities of Ribosome-Bound Nascent Polypeptides Probed by Mass Spectrometry. *Proc. Natl. Acad. Sci.* **2023**, *120* (33), e2303167120. https://doi.org/10.1073/pnas.2303167120.

(2) Tan, R.; Hoare, M.; Bellomio, P.; Broas, S.; Camacho, K.; Swovick, K.; Welle, K. A.; Hryhorenko, J. R.; Ghaemmaghami, S. Formylation Facilitates the Reduction of Oxidized Initiator Methionines. bioRxiv February 7, 2024, p 2024.02.06.579201. https://doi.org/10.1101/2024.02.06.579201.

(3) Hoare, M.; Tan, R.; Welle, K. A.; Swovick, K.; Hryhorenko, J. R.; Ghaemmaghami, S. Methionine Alkylation as an Approach to Quantify Methionine Oxidation Using Mass Spectrometry. *J. Am. Soc. Mass Spectrom.* **2024**, *35* (3), 433–440. https://doi.org/10.1021/jasms.3c00337.

(4) Chambers, M. C.; Maclean, B.; Burke, R.; Amodei, D.; Ruderman, D. L.; Neumann, S.; Gatto, L.; Fischer, B.; Pratt, B.; Egertson, J.; Hoff, K.; Kessner, D.; Tasman, N.; Shulman, N.; Frewen, B.; Baker, T. A.; Brusniak, M.-Y.; Paulse, C.; Creasy, D.; Flashner, L.; Kani, K.; Moulding, C.; Seymour, S. L.; Nuwaysir, L. M.; Lefebvre, B.; Kuhlmann, F.; Roark, J.; Rainer, P.; Detlev, S.; Hemenway, T.; Huhmer, A.; Langridge, J.; Connolly, B.; Chadick, T.; Holly, K.; Eckels, J.; Deutsch, E. W.; Moritz, R. L.; Katz, J. E.; Agus, D. B.; MacCoss, M.; Tabb, D. L.; Mallick, P. A Cross-Platform Toolkit for Mass Spectrometry and Proteomics. *Nat. Biotechnol.* **2012**, *30* (10), 918–920. https://doi.org/10.1038/nbt.2377.
